# Biological impact and therapeutic implication of tumor-associated macrophages in hepatocellular carcinoma

**DOI:** 10.1038/s41419-024-06888-z

**Published:** 2024-07-12

**Authors:** Deming Li, Ting Zhang, Ye Guo, Cong Bi, Ming Liu, Gang Wang

**Affiliations:** 1 Department of Anesthesiology, The Fourth Affiliated Hospital of China Medical University, Shenyang, 110032 PR China; 2 Department of Surgical Oncology and General Surgery, The First Hospital of China Medical University, Shenyang, 110001 PR China; 3 Department of Intervention, The Fourth Affiliated Hospital of China Medical University, Shenyang, 110032 PR China; 4 Department of Radiology, The First Hospital of China Medical University, Shenyang, 110001 PR China; 5 Department of Oral Radiology, School of Stomatology, China Medical University, Shenyang, Liaoning 110002 PR China

**Keywords:** Cancer epigenetics, Cancer metabolism

## Abstract

The tumor microenvironment is a complex space comprised of normal, cancer and immune cells. The macrophages are considered as the most abundant immune cells in tumor microenvironment and their function in tumorigenesis is interesting. Macrophages can be present as M1 and M2 polarization that show anti-cancer and oncogenic activities, respectively. Tumor-associated macrophages (TAMs) mainly have M2 polarization and they increase tumorigenesis due to secretion of factors, cytokines and affecting molecular pathways. Hepatocellular carcinoma (HCC) is among predominant tumors of liver that in spite of understanding its pathogenesis, the role of tumor microenvironment in its progression still requires more attention. The presence of TAMs in HCC causes an increase in growth and invasion of HCC cells and one of the reasons is induction of glycolysis that such metabolic reprogramming makes HCC distinct from normal cells and promotes its malignancy. Since M2 polarization of TAMs stimulates tumorigenesis in HCC, molecular networks regulating M2 to M1 conversion have been highlighted and moreover, drugs and compounds with the ability of targeting TAMs and suppressing their M2 phenotypes or at least their tumorigenesis activity have been utilized. TAMs increase aggressive behavior and biological functions of HCC cells that can result in development of therapy resistance. Macrophages can provide cell–cell communication in HCC by secreting exosomes having various types of biomolecules that transfer among cells and change their activity. Finally, non-coding RNA transcripts can mainly affect polarization of TAMs in HCC.

## Facts

- Tumor-associated macrophages (TAMs) play a significant role in the tumor microenvironment of hepatocellular carcinoma (HCC), with a predominant M2 polarization contributing to tumor progression.
- The presence of TAMs in HCC correlates with increased tumor growth, invasion and induction of glycolysis, leading to the distinct metabolic reprogramming of HCC cells and promoting malignancy.
- Molecular networks regulating the conversion of TAMs from M2 to M1 polarization have been identified as potential targets for therapeutic intervention in HCC.
- Various drugs and compounds targeting TAMs and suppressing their M2 phenotypes or tumorigenesis activity have been explored as potential therapeutic strategies for HCC.
- Macrophages facilitate cell–cell communication in HCC through the secretion of exosomes containing various biomolecules that modulate the activity of neighboring cells, contributing to tumor progression and therapy resistance.

## Open questions

- What specific factors or signaling pathways drive the polarization of TAMs toward the M2 phenotype in the context of HCC, and how can these pathways be effectively targeted to skew TAM polarization toward an anti-tumorigenic M1 phenotype?
- How do the interactions between TAMs and HCC cells modulate the tumor microenvironment to promote tumor growth, invasion and therapy resistance, and what are the key mediators of these interactions?
- What are the mechanisms underlying the induction of glycolysis in HCC cells by TAMs, and how can metabolic reprogramming be exploited as a therapeutic target in HCC treatment?
- What is the role of non-coding RNA transcripts in regulating the polarization of TAMs in HCC, and how can targeting these transcripts offer novel therapeutic approaches for HCC management?
- What are the potential limitations and challenges associated with targeting TAMs as a therapeutic strategy in HCC, and how can these obstacles be overcome to improve treatment efficacy and patient outcomes?

## Introduction

Hepatocellular carcinoma (HCC) is dominant type (80%) of primary liver cancer that is considered the fifth most common tumor around the world and up to 500,000 new cases are diagnosed annually [1, 2]. The incidence rate of HCC is different in males and females; so that, it is the second leading cause of death in males and its occurrence is lower in females and is the sixth leading cause of death in women. Although HCC occurs in various regions in the world, China shows highest number of cases (50%) and it has a record of highest number of deaths [3, 4]. One of the mechanisms led to death in HCC patients and therapy failure is metastasis [5]. Unfortunately, metastasis and recurrence rate of HCC can reach to 50% that is a troublesome problem [6, 7]. Hepatectomy and liver transplantation are current therapies used for the treatment of HCC, but as it was mentioned, the efficacy of these treatments is compromised by recurrence and invasion of tumor cells [8]. The metastasis and recurrence in HCC depend on a number of special cells known as circulating tumor cells (CTCs) that are separated from primary tumor site and obtain ability to enter into peripheral blood [9, 10]. Therefore, when clinicians are following the process of HCC therapy, they should be careful about the treatment strategy that they use and by using novel therapeutics, it is better to target CTCs in effective cancer therapy. When primary liver tumors are developed, there are hepatocyte injuries and large-scale experiments have highlighted not only the mechanisms participating in HCC invasion and recurrence but also conventional and modern therapies for HCC such as targeted immunotherapy, surgical resection, systemic chemotherapy and others [11–16].

**Fig. 1:**
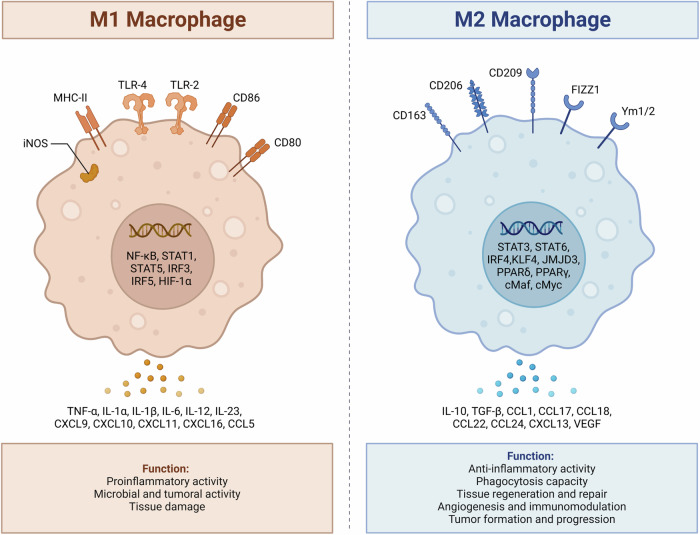
Macrophages can be found in two phenotypes including M1 and M2. The M2 polarized macrophages are characterized with the expression of a number of receptors including CD163, CD206, CD209, FIZZ1 and Ym1/2 that can secrete different cytokines including CCL22. The M1 polarized macrophages are characterized with the expression of a number of receptors including iNOS, MHC-II, TLR-4, TLR-2, CD86 and CD80 that can secrete TNF-α, IL-1α and others. In cancer, the M2 polarized macrophages increase tumorigenesis, while the M1 polarized macrophages reduce cancer progression (created by Biorender.com).

One of the reasons for the inability in HCC treatment is complicated interactions observed in tumor microenvironment (TME) that provide an optimal condition for the progression of cancer cells. In the TME, there is competition for the proliferation of tumor cells and this can lead to reduction in oxygen levels. When hypoxia occurs in TME, it leads to overexpression of HIF-1α to enhance levels of LOXL2 in the generation of vasculogenic mimicry and increasing HCC progression [17]. When hypoxia is present in TME, it increases the levels of MYDGF and then angiogenesis occurs, which reshapes TME for increasing the growth rate of HCC cells [18]. Sometimes, the interactions of TME can compromise the efficacy of immunotherapy in HCC. After HCC chemotherapy with sorafenib, CXCR4 was suppressed and this avoided the formation of an immunosuppressive TME, suppressed cancer proliferation and diminished invasion of tumor cells to lung. Furthermore, suppression of CXCR4 in TME is beneficial in improving the potential of anti-PD-L1 immunotherapy in HCC [19]. Importantly, the exosomes secreted by HCC cells can participate in the remodeling of TME and affecting progression of tumor cells. Exosomal miR-761 can be secreted by HCC cells and by increasing levels of JAK2 and STAT3, it participates in affecting TME and these exosomes are absorbed by normal fibroblasts [20]. CAPS1 demonstrates association with some alterations in TME and when expression level of CAPS1 reduces in HCC cells, it leads to malignant behavior of cancer cells, advanced stage and unfavorable prognosis of patients. Moreover, increasing the expression level of CAPS1 suppresses growth and invasion of HCC cells via changing exocytosis-associated TME [21]. Immune checkpoint blockade is also an important therapy for HCC, but when changes occur in TME, the efficacy of this therapy significantly reduces. MTDH is able to enhance levels of PD-L1 via β-catenin/LEF-1 to compromise immunotherapy. However, when expression of MTDH is suppressed, it promotes infiltration of cytotoxic T cells in TME and sensitizes HCC cells to checkpoint blockade therapy (anti-PD-1 therapy) [22]. According to these studies, alterations in TME of HCC cause significant changes in the progression of tumor cells [23, 24]. Therefore, the current review focuses on the role of macrophages in TME and function of these immune cells in affecting progression of tumor cells via providing complex interactions. Figure 1 demonstrates the different polarization of macrophages.

## Origin of TAMs in HCC

The source of tumor-associated macrophages (TAMs) is heterogeneous [25]. There is evidence that both macrophages that reside in tissues (such as the liver’s Kupffer cells (KCs)) and those that originate from monocytes drawn from the blood play a role in the development of TME [26, 27]. However, the main source of TAMs, according to the hypothesis, is circulating blood monocytes [28]. In mice, TAMs primarily originate from bone marrow (BM) monocytes. In human HCC, TAMs stem from CCR2+ monocytes. These monocytes are attracted by inflammatory signals emitted by cancer cells in both primary and metastatic tumors. Under the influence of chemokines and growth factors secreted by stromal and tumor cells, these monocytes can differentiate into TAMs, subsequently aiding in tumor progression [28–31]. For example, an increase in TAMs induced by lysyl oxidase-like 4 (LOXL4: a copper-dependent monoamine oxidase in the extracellular matrix) in mice is primarily attributable to monocyte infiltration; LOXL4 is capable of suppressing the proliferation of resident liver macrophages and nearly eliminating them during the development of HCC [32]. Additionally, according to current research, tumors in chronically damaged liver tissues attract monocyte-derived macrophages more often than tumors in healthy livers [33]. Another critical process for the buildup of tumor-infiltrating macrophages in HCC tissues is self-replication [34]. It should be mentioned that there is evidence that macrophages in the liver can be initiated by cells derived from the yolk sac and fetal liver. These cells can then undergo self-renewal and be supplied with monocytes [35–37]. For instance, Ye et al. found that the predominant TAMs in orthotopic HCC in RBPj cKO mice showed characteristics of KCs, indicating that TAMs in situ in HCC probably come from embryonic hematopoiesis-generated KCs and BM monocyte-derived KCs, which are thought of as self-renewing tissue-resident macrophages [38–40]. However, the exact source of these KC-like TAMs is not yet known; possible candidates include monocyte-derived TAMs, mononuclear cells sourced from BM or extramedullary locations, or even actual KCs. Although KCs may constitute a tiny fraction of the overall TAM pool in HCC, KC-like TAMs discriminate from actual KCs, BM-derived monocytes and extramedullary monocytes [38].

## Polarization of macrophages in HCC

As mentioned earlier, the significant changes that occur during the progression of HCC in TME can increase the potential of tumor cells in progression and promote the pathogenesis of the disease. In fact, one of the most abundant cells in TME is macrophages and due to their crosstalk with other cells such as tumor cells and immune cells in TME, it is essential that macrophages play a significant role in tumorigenesis in HCC. The aim of current section is to understand the mechanisms that can mediate M2 polarization of macrophages. In this case, the pathways modulating macrophage polarization can be categorized into oncogenic and onco-suppressor factors. FNDC5 is responsible for changing white adipose tissue to brown adipose tissue and it is secreted during exercise that modulates energy metabolism in vivo [41]. When protease hydrolysis occurs, FNDC5 is able to release irisin into tissues and it shows vital functions in pancreas, liver, brain, heart and testis, among others [42]. FNDC5 has been considered as an oncogenic factor and upregulation of FNDC5 can result in epithelial–mesenchymal transition (EMT) in enhancing tumor invasion [43]. Moreover, overexpression of irisin/FNDC5 is observed in HCC [44]. FNDC5 can affect M2 polarization of macrophages in TME of HCC and in this way, it needs to affect various factors to coordinate them in making such alterations in TME. On the surface of HCC cells, there is FNDC5 that after hydrolysis converts into irisin for affecting macrophages. Irisin increases PPARγ levels to stimulate M2 polarization. Moreover, upregulated PPARγ inhibits NF-κB and NLRP3 expression levels in mediating M1 polarization of macrophages in decreasing HCC progression. Therefore, function of FNDC5 in enhancing HCC progression is based on triggering M2 polarization of macrophages and if the interaction of FNDC5 with other pathways is suppressed, it can lead to M1 polarization and reduced progression of tumor cells [45]. One of the problems is dual function of molecular pathways regulating macrophage polarization in HCC. OIT3 elevates levels of ALOX15 and CYP4F3 to promote ROS generation in mediating ferroptosis and disrupting HCC progression [46]. However, OIT3 has been displayed to increase HCC malignancy via affecting macrophage polarization in another experiment. The immune characteristics in HCC and metabolic reprogramming in M2 polarized macrophages are modulated by OIT3. High expression of OIT3 leads to M2 polarization of macrophages and can enhance metastasis of tumor cells [47].

Accumulating data highlight the fact that crosstalk that is present between tumor cells and macrophages can produce a force for increase in progression of tumors [48]. On the other hand, the cell–cell interactions can be regulated by a number of soluble factors such as Wnt [49]. The Wnt ligands are considered as secreted proteins that can modulate proliferation, migration and tissue remodeling during embryogenesis, and their critical function during cancer progression has also been clarified [50–52]. Wntless is able to modulate the secretion of Wnt ligands into extracellular environment and after binding to Frizzled receptors on the surface of cells, they stimulate Wnt/β-catenin pathway [53]. In M2 polarization of macrophages and during the process of monocyte to macrophage differentiation, upregulation of Wnt is observed. When Wnt silencing occurs in macrophages, the anti-tumor activity is observed. However, increase in expression level of Wnt can result in upregulation of c-Myc in mediating M2 polarization of macrophages. Such function of Wnt on macrophages can significantly increase growth and invasion of HCC cells [54]. Increase in M2 polarization of macrophages can be obtained by ZIP9 and this transcription factor decreases M1 polarization of macrophages in increasing HCC malignancy [55].

There are differences in function of SLAMF6 among human cancers in regulating tumorigenesis. Loss of SLAMF6 can enhance potential of cancer elimination and it is defined as a T cell checkpoint modulator [56]. Furthermore, SLAMF6 can improve function of CD8+ T cells in exerting anti-cancer activity against melanoma [57]. In HCC, SLAMF6 can increase Ly108 levels to induce NF-κB in mediating M2 polarization of macrophages and increasing tumorigenesis. Silencing Ly108 leads to inhibition of NF-κB and reduction in M2 polarization of macrophages that is against progression of HCC [58]. During polarization of macrophages from M2 phenotype to M1, the expression level of lnc-Ma301 significantly enhances, while lnc-Ma301 displays low expression in HCC cells and tissues. Lnc-Ma301 interacts with caprin-1 protein to suppress proliferation and metastasis of HCC cells, and to inhibit EMT via reducing Slug and vimentin levels, and increasing E-cadherin levels. Moreover, lnc-Ma301 and caprin-1 interaction can suppress Akt/Erk1 axis in reducing HCC progression [59]. This experiment provides the fact that when a factor, that is responsible for regulating macrophage polarization, is identified in HCC, more investigation can be performed in understanding its function in affecting other pathways in modulating carcinogenesis.

**Fig. 2:**
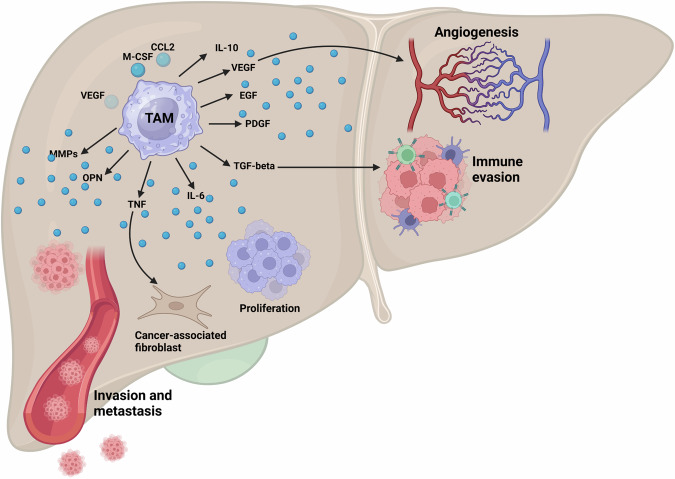
The critical function of macrophages in HCC [151]. The TAMs are able to secrete different kinds of secretions to enhance metastasis and proliferation of tumor cells. They can activate cancer-associated fibroblasts. Moreover, the induction of angiogenesis and promotion of immune evasion can be mediated by TAMs (created by Biorender.com).

Cyclooxygenase-2 (COX-2) displays association with formation of TME and can lead to unfavorable prognosis [60]. Suppressing acetylation of COX-2 in mitochondria with resveratrol can decrease growth and mitochondrial fission for promoting drug sensitivity in HCC [61]. Furthermore, the presence of a loop between COX-2 and yes-associated protein (YAP) can increase the growth and progression of HCC [62]. When expression level of COX-2 enhances, it causes M2 polarization of macrophages to trigger exhaustion in T cytotoxic cells in increasing HCC progression [63]. As a metabolic enzyme, CYP2A6 has shown potential in regulating HCC progression, which exerts its function by increasing the M2 polarization of macrophages. However, TSIIA suppresses CYP2A6 to increase M1 polarization of macrophages in HCC suppression [64]. PCSK9 is one of the factors whose function in cancer is mutual and it can be oncogenic or an onco-suppressor. For instance, pseurotin A prevents secretion of PCSK9 and its association with LDL receptor in reducing breast tumor malignancy [65]. Moreover, PCSK9 expression increases by *Actinidia chinesis* Planch root extract in suppressing cholesterol metabolism in HCC [66]. PCSK9 reduces apoptosis in HCC to enhance proliferation rate of tumor cells [67]. PCSK9 shows interaction with polarization of macrophages in HCC that increases levels and secretion of OX40L from HCC cells in disrupting M2 polarization of macrophages and impairing tumorigenesis [68]. Therefore, polarization of macrophages in HCC is modulated by different factors such that each experiment provides a unique pathway in this case [69–72]. Figure 2 highlights the function of TAMs in HCC.

## Macrophages in the pathogenesis of HCC

The increasing evidences have highlighted the fact that macrophages play a significant role in the pathogenesis of HCC [73]. The M2 polarized macrophages contribute to fatty acid oxidation (FAO)-related mechanisms in HCC pathogenesis. The pro-inflammatory function of M2 macrophages can be increased by IL-1β, while the release of IL-1β secretion increases by FAO relying on ROS and NLRP3 inflammasome [74]. In order to better understand the mechanism of HCC pathogenesis, the various models have been developed. Schneider et al. have shown that DEN-mediated hepatocarcinogenesis can mediate liver inflammation with an increase in intrahepatic levels of macrophages and cytotoxic T cells. Notably, the enhancement in the accumulation of macrophages in chemokine scavenger receptor D6-deficient mice has no effect on the pathogenesis of HCC [75]. The release of IL-6 macrophages is a potential factor in the tumorigenesis [76]. In HCC, there is an increase in M2 macrophage infiltration in TME of HCC cells that secrete IL-17 in tumorigenesis [77]. The pathogenesis of HCC can be accelerated through the function of TAMs in the secretion of exosomes transferring CD11b/CD18 (integrin αMβ2) [78]. Macrophages emanate from the monocytes circulating in the bloodstream. The upregulation of CCL2/CCR2 is vital for the monocyte recruitment to transform into macrophages that can be transformed into M2 polarized macrophages for impairing CD8+ T cell-induced anti-cancer immune responses [31]. The TAMs have been shown to secrete IL-1β in hypoxic TME that demonstrated upregulation of HIF-1α. Moreover, the presence of necrotic HCC cells enhances the release of IL-1β by M2 polarized macrophages through induction of TLR-4/TRIF/NF-κB axis [79]. The interactions of CD48/2B4 induce peritumoral macrophage infiltration to reduce the activity of natural killer cells for HCC pathogenesis [80]. Notably, the liver CD14+ inflammatory macrophages can respond to infected hepatocytes during chronic HBV and are able to release IL-23 for favoring HCC [81].

## Macrophages and increase in HCC progression (proliferation and metastasis)

The aim of current section is to evaluate the role of macrophages in regulating the growth and invasion of HCC cells. The reason of focusing on both proliferation and invasion is that studies have investigated them together; therefore, it is better to combine them in this section. The macrophages with pro-tumoral activity are able to increase both growth and invasion of HCC cells. The presence of hypoxia in TME leads to upregulation of HIF-1α that stimulates glycolysis for increasing CA12 levels to avoid apoptosis in macrophages. Moreover, glycolysis leads to increase in levels of cytokines including IL-10, IL-1β and TNF-α for preventing apoptosis in macrophages and increasing growth and invasion of HCC cells [82]. On the other hand, the M2 polarization of macrophages can lead to changes in molecular pathways for HCC malignancy and accelerating carcinogenesis. Macrophages are able to release extracellular vesicles (EVs) containing miR-17-92 cluster to stimulate imbalance in TGF-β1/BMP-7 by enhancing TGFBR2 levels at post-transcriptional level and suppressing ubiquitylation of ACVR1. Then, expression level of ID1 enhances to trigger HCC proliferation and invasion [83]. The dihydroartemisinin has been shown to suppress PI3K/Akt axis and decreases fibronectin 1 (FN1) and integrin-β1 in impairing tumorigenesis in HCC [84]. PRPF8 changes FN1 splicing and stimulates FAK/Akt axis in increasing HCC progression [85]. Therefore, function of FN1 is in favor of HCC tumorigenesis. TAMs are able to secrete FN1 that increases TNFRSF11B levels to upregulate SMAD3. Then, stimulation of JUN occurs that promotes metastasis of HCC via inducing EMT [86].

The stability of SIX1 can be increased by O-GlcNAcylation that causes significant enhancement in growth rate of HCC cells [87]. Moreover, degradation of SIX1 and RPS16 can increase sorafenib sensitivity in HCC [88]. The ability of macrophages in promoting HCC invasion can be related to increasing MMP-9 expression. Macrophages promote SIX1 levels to upregulate p65. Then, p65 follows two ways (indirect and direct methods) to enhance cancer invasion. In direct way, p65 promotes MMP-9 expression; in indirect way, p65 promotes IL-6 secretion that induces STAT3 axis in promoting MMP-9 levels and enhancing invasion and metastasis of HCC cells [89]. Interestingly, the interaction of some cells in TME can affect polarization of macrophages in modulating HCC progression. Cancer-associated fibroblasts (CAFs) demonstrate high infiltration in TME of HCC. CAFs are able to reduce exosomal levels of miR-150-3p in HCC progression [90] and mediate immunotherapy resistance [91]. The CAFs in TME can secrete CXCL12 that binds to CXCR4 on the surface of TAMs to induce their M2 polarization for enhancing proliferation, metastasis and EMT in HCC cells [92].

Another factor that modulates HCC progression is Tim-3, and its inhibition can improve function of lymphocytes in HCC therapy [93]. NEAT1 promotes Tim-3 expression by miR-155 suppression in increasing HCC progression [94]. HCC cells secrete TGF-β that binds to promoter of Tim-3 in enhancing its expression and inducing M2 polarization of macrophages for promoting invasion and proliferation [95]. One of the reasons for HCC progression is bacterial infection, but mechanism of action is completely unique. Pathogenic bacteria can cause secretion of IL-25 from tuft cells and then, IL-26 circulates in bloodstream to induce M2 polarization of macrophages. Then, these macrophages secrete CXCL10 to enhance carcinogenesis and metastasis of HCC cells [96]. Moreover, the presence of alveolar macrophages leads to upregulation and production of Leukotriene B4 in increasing lung invasion of HCC cells [97]. According to these studies, changes in molecular pathways and their critical interactions with macrophages can result in alterations in HCC progression and when M2 polarization of macrophages is induced, it promotes tumorigenesis [98–102].

## Macrophages and glycolysis in HCC

Aerobic glycolysis or Warburg effect is one of the important hallmarks of cancer that involves transformation of glucose into lactate. Although the efficacy of glycolysis in ATP generation is low and under question, but it brings immediate energy and nutrients for tumor growth [103–105]. Recent studies have focused on glycolysis in HCC and it appears that it shows dysregulation in tumor cells. CircPRN2 suppresses miR-183-5p expression to inhibit glycolysis in HCC cells [106]. Moreover, upregulation of SLC25A51 results in increase in SIRT5 expression in accelerating glycolysis in HCC cells [107]. Hence, various signaling networks and interactions modulate glycolysis mechanism in HCC [108, 109] and the current section briefly focuses on macrophage and glycolysis interaction in HCC. First of all, interaction of HCC cells and macrophages is vital for glycolysis mechanism regulation in HCC cells. Macrophages in TME can be educated by HCC cells in inducing glycolysis. HCC cells secrete IL-10 and TGF-β to educate macrophages that, in turn, increase nuclear transfer of β-catenin in increasing c-Myc levels, leading to glycolysis and enhancement in proliferation and metabolism of HCC cells. Notably, this process leads to M2 polarization of macrophages, which, in turn, induce metastasis and EMT by increasing HCC progression and promote their potential in education of macrophages [110]. When loss of dectin 3 occurs in HCC, it leads to glycolysis induction in macrophages that enhances proliferation of HCC cells and avoids apoptosis [111]. Furthermore, arsenic trioxide stimulates AMPK signaling in facilitating glycolysis in macrophages [112]. However, a few studies have focused on macrophage and glycolysis in HCC and there is still much space for investigation.

## Macrophages and immunity in HCC

**Fig. 3:**
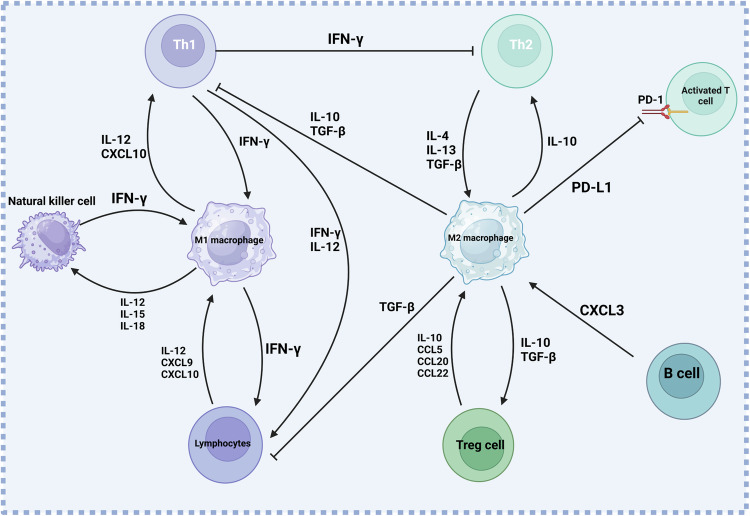
The interaction of macrophages with immune cells in HCC [255]. The M1 macrophages increase the function of immune cells such as Th1 cells, lymphocytes and natural killer cells through the secretion of various factors including IFN-γ, ILs and CXCLs, while M2 polarized macrophages impair the function of T cells and lymphocytes, and they induce Treg cells, further highlighting the role of macrophages in the regulation of immune system (created by Biorender.com).

The immune system is a complex network responsible for the suppression of tumor [113]. The host defense can be mediated by macrophages and they participate in the innate and adaptive immune reactions to pathogens. Moreover, macrophages can regulate inflammatory responses. The exposure of macrophages into the different anti-inflammatory factors including TGF-β1, IL-10, IL-4, and prostaglandin E2 can lead to M2 polarization of macrophages [13]. The predominant kind of macrophages in tumors is M2 and they can be characterized by the poor expression of differentiation-related macrophage antigens including CD51 and carboxypeptidase M, high constitutive expressions of IL-10, IL-6, IL-1ra, IL-1 decoy and arginase I, downregulation of TNF and IL-12. Moreover, the M2 polarized macrophages can secrete chemokines including CCL17 and CCL22 [8, 27, 28]. The macrophages can determine the response of HCC cells to immunotherapy. The loss of GSK3β in macrophages can reduce the tumorigenesis in HCC and this is also of importance for improving the sensitivity of cancer cells to PD-1 blockade immunotherapy [114]. This has also been approved in another study showing that inhibition of CacyBP not only prevents the recruitment of macrophages, but also improves the function of PD-1 blockage in cancer immunotherapy [115]. The TAMs can mediate immunosuppression in HCC. CKI is able to enhance pro-inflammatory responses and decreased the TAM-induced immunosuppression in HCC through increasing TNFR1-induced NF-κB and p38 MAPK axis. CKI-primed macrophages have been shown to enhance the function of CD8+ T cells and diminish their exhaustion in enhancing apoptosis in HCC [116]. Noteworthy, bufalin increases M1 polarization of macrophages in enhancing anti-cancer immune reactions in HCC [117]. Upon the M2 polarization of macrophages, they secrete EVs in enhancing nuclear transfer of IQGAP1 and upregulate STAT3 phosphorylation via MISP inhibition to induce immune evasion [118]. It is worth mentioning that induction of M2 polarization of macrophages by COX-2 expression can cause exhaustion of T cells [63]. Regarding the importance of macrophages in the HCC immunity, there have been efforts for the regulation of macrophage polarization. The HCC vaccine developed based on *Listeria* can upregulate NF-κB via TLR-2 and MyD88 overexpression, and it mediates p62 recruitment to induce the autophagy axis, causing M1 polarization of macrophages in sensitizing HCC cells to anti-PD-1 therapy [119]. Figure 3 demonstrates the interaction of macrophages with immune cells in HCC.

## Macrophages and therapy response in HCC

The progression of HCC cells is an aspect and another part is role of macrophages in TME in regulating therapy response in tumor cells. It has been obvious for researchers that chemoresistance in HCC is not only related to pre-clinical studies but also that, based on the clinical results and therapy failures, chemoresistance commonly occurs at the clinical level and it is much worthy of investigation. Although the role of macrophages in TME in mediating therapy resistance in HCC has been a little ignored, there are enough evidences showing that macrophages are potent regulators of therapy response in HCC. Sorafenib is a multi-kinase suppressor and is the only drug that has been clinically confirmed for treatment of cancer patients in advanced stages [120]. The anti-tumor function of sorafenib is based on suppressing Raf and the kinase activity of vascular endothelial growth factor receptor and platelet-derived growth factor receptor [121]. Although advantageous of sorafenib in patients has been approved, the response of tumor cells is low and only a mild increase in survival rate of patients is observed [122]. One of the most important reasons is the development of resistance to sorafenib chemotherapy in HCC. Tumor-associated neutrophils have been able to recruit macrophage and T-regulatory cells to stimulate resistance to sorafenib in HCC [123]. This experiment clearly reveals that increase in progression of HCC can be provided by changes in macrophages in TME that can finally lead to development of drug resistance. When it comes to evaluating the underlying mechanisms involved in tumorigenesis, it is shown that when macrophages infiltrate into TME, some changes occur in molecular pathways that affect chemotherapy response in HCC. CXCR2 is an oncogenic factor that mediates poor prognosis in HCC [124] and its downregulation by miR-940 results in reduction in metastasis of tumor cells [125]. TAMs are able to increase progression of HCC and for this purpose, they induce CXCR2 signaling to mediate sorafenib resistance in HCC [126]. When TAMs infiltrate in TME, they secrete HGF that leads to stimulation of PI3K/Akt, MAPK and c-Met pathways in increasing progression of HCC cells, resulting in sorafenib resistance [127]. However, sorafenib is not the only chemotherapy drug in HCC therapy and based on the studies, oxaliplatin can also be used for HCC treatment. One of the mechanisms that can regulate therapy response in tumor cells is autophagy [128–130]. TAMs present in TME, stimulate autophagy mechanism to inhibit apoptosis in HCC cells and mediate oxaliplatin resistance [131]. However, the role of TMAs in mediating resistance to other chemotherapy agents, such as doxorubicin and paclitaxel, should be evaluated. Moreover, the role of TAMs in inducing radio-resistance in HCC cells should be investigated in near future.

## Macrophages and exosome interaction in HCC

It was discussed that interaction of macrophages and tumor cells in TME can affect biological behavior of cancer cells. The intercellular crosstalk occurring in TME plays a significant role in tumorigenesis. One of the most well-known classical ways for crosstalk is cell–cell contact and direct secretion of soluble factors. Exosomes are considered as small structures and an appropriate method for intercellular signal delivery system during cancer malignancy. Exosomes are considered as nanoscale vesicles that they contain lipid bilayer membranes with ability of containing proteins, lipids and nucleic acids, derived from multivesicular bodies (MVBs) [132–135]. The TME remodeling can be achieved by exosomes through providing communication of cancer cells and stromal cells by exosomes [136]. There are interactions between exosomes and macrophages in TME of HCC that are discussed in current section. The inhibition of CD8+ T cell activity in HCC can aggravate tumorigenesis [137]. When suppression of PD-L1 occurs, the function of glycolytic macrophages in HCC inhibition increases [138]. When GOLM1 expression increases during HCC progression, it causes exosomal delivery of PD-L1 to TAMs to mediate disrupting function of CD8+ T cells in accelerating HCC progression [137]. On the other hand, it has been reported that macrophages have ability of secreting exosomes in regulating HCC progression. The TAMs secrete exosomes with such high levels of lncMMPA that, due to its oncogenic function, it reduces miR-548s’ expression to increase ALDH1A3 levels in glycolysis induction, mediating tumor metabolism and accelerating the progression of HCC [139]. Although it is not the scope of current review, but it is worth mentioning that exosomes can be engineered in laboratory for purpose of cancer therapy and such approach has been followed in HCC treatment. PIONs-contained exosomes have been shown to increase M1 polarization of macrophages in HCC and are able to suppress cancer progression in vivo [140]. Based on the studies, exosomes are able to regulate polarization of macrophages in HCC progression regulation. Sometimes, exosomes do not change polarization of macrophages and they focus on the inducing cell death in M1 polarized macrophages to eliminate their suppressive effect and increase HCC progression. The liver cancer cells that have been infected by HBV, secrete exosomes containing miR-142-3p to stimulate ferroptosis in M1 polarized macrophages in enhancing liver tumor progression [141]. It appears that infection with HBV is a way to regulate secretion of exosomes in HCC. The HCC cells infected with HBV can secrete exosomal miR-142-3p to reduce SLC3A2 levels in inducing ferroptosis in M1 polarized macrophages and elevating HCC malignancy [142].

The function of non-coding RNA (ncRNA) transcripts in regulating HCC progression by affecting macrophages is discussed in detail in next section, but most of the studies, that have evaluated interaction of exosomes and macrophages in HCC, have focused on exosomal ncRNAs. Exosomal hsa_circ_0004658 can be secreted by macrophages that are abundantly present in TME of HCC and one of the interesting points is ability of such exosomes in reducing HCC malignancy, showing that exosome secretion by macrophages does not always leads to HCC progression. Exosomal hsa_circ_0004658 increases JAM3 expression by miR-499b-5p sponging to suppress malignancy of HCC [43]. When the liver tumor cells exposed to various stresses and stimuli such as endoplasmic reticulum stress, it causes release of exosomes containing miR-23a-3p that suppresses PTEN signaling to increase Akt levels. Then, upregulation of PD-L1 occurs in macrophages that these cells reduce number of CD8+ T cells and IL-2 levels in TME and promote apoptosis in reducing HCC progression [143]. Moreover, when M1 polarized macrophages secrete exosomes, it can lead to disruption of molecular pathways related to HCC malignancy. The M1 polarized macrophages secrete exosomes comprising of miR-628-5p that prevents m6A modification of circFUT8 in reducing progression of HCC cells [144]. Overall, exosomes can be secreted from macrophages to regulate biological behavior of HCC cells or it can be derived from HCC cells and other kinds of cells that final impact will be on the progression of tumor cells and based on these studies, exosomes can transfer important bioactive molecules and provide interaction and crosstalk of macrophages and tumor cells in TME that modulating their biogenesis and secretion can lead to development of novel therapeutics for HCC in future [145–149].

## Epigenetic changes and macrophages in HCC

### Non-coding RNAs

Both macrophages and ncRNAs play crucial roles in tumor development and progression [150]. Both tumor cells and macrophages are able to communicate with one another and share information and materials via cell-to-cell contact, which can be facilitated by costimulation, produced cytokines, or EVs such as microvesicles and exosomes [151–156]. Among these, upon fusion of MVBs, the tiniest vesicles, known as exosomes, are released. Their diameter ranges from 30 to 150 nm [157]. By transmitting data via their cargo, they promote communication between tumor cells and their surroundings and prevent the breakdown of information molecules such as proteins, DNA and ncRNAs [158–160]. Additionally, the interplay between macrophages and tumor cells, facilitated by these costimulatory molecules, cytokines and EVs, can lead to the progression or regression of malignancies [153, 160]. In recent years, more and more research has shown that ncRNAs can be secreted in exosomes or microvesicles from tumor cells and macrophages alike [161, 162]. These ncRNAs have a significant role in regulating the interaction between tumor cells and macrophages because of their well-documented roles in tumor cell and macrophage biology. On the one hand, as indicated before, ncRNAs play a role in tumor cell proliferation, invasion, migration, apoptosis or treatment resistance by acting as either tumor suppressors or oncogenes. On the other hand, they have a role in regulating the infiltration, recruitment, activation or polarization of macrophages [163]. For example, key players in macrophage activation and M1 polarization include miR-155, lncRNA CCAT1 and miR-34-a [164–167]. Conversely, lncRNA MALAT1, miR-21 and miR-Let-7 at high levels promote M2 polarization in macrophages [168–171]. Moreover, it has been shown that modulating the phenotype and function of macrophages mediates the regulation of ncRNAs in tumor cells [161, 172]. Thus, ncRNAs can influence cancer growth and progression through mediating the interaction between tumor cells and macrophages. This is true regardless of whether the ncRNAs originate from tumor cells or macrophages, are self-expressed, or are produced from cell-derived EVs.

The covalently closed loop structures lacking classic 5′ caps or 3′ polyadenylated tails are known as circular RNAs (circRNAs) that are generated through back-splicing process [173]. CircRNAs demonstrate critical function in modulating HCC progression and their interaction with macrophages has been evaluated in a number of studies. Circ-0110102 reduces miR-580-5p expression to downregulate PPARα in decreasing secretion and levels of CCL2. Then, inhibition of p38 MAPK/Foxo1 occurs to reduce COX-2 and PEG levels in suppressing macrophages and reducing HCC progression [174]. However, the function of hsa_circ_0003410 is different and it causes increase in HCC tumorigenesis. The process of back-splicing in UBAP2 leads to upregulation of hsa_circ_0003410 to increase secretion of CCL5 protein for binding to CCR5 on the macrophages in inducing M2 polarization of macrophages and increasing HCC malignancy [175].

### DNA methylation

Recent research has linked DNA methylation regulation of gene promoters or enhancers to dysfunctional gene expression in liver disorders, such as HCC [176]. The importance of changed DNA methylation in remodulating the TME of HCC through affecting macrophage infiltration and differentiation is becoming more and more demonstrated. Poor clinical outcomes in liver cancer patients are predicted by a correlation between the infiltration level of macrophages and centrosomal protein 55 overexpression, which may be aided by DNA hypomethylation [177]. Elevated levels of methylated CpG sites in the angiopoietin-like 4 (ANGPTL4) promoter, which are strongly linked to advanced tumor stage, lead to ANGPTL4 deregulation in HCC [178]. By reducing tumor-favorable microenvironment factors such as CD68+ macrophage infiltration and changes to the profile of cytokines released by macrophages in the TME, Ad-ANGPTL4 treatment considerably reduced the development of HCC.[178]. Novel molecular targets for HCC have been suggested to be macrophage-related chemokines, such as CXCL2. It is still debatable, nevertheless, how CXCL2 is expressed. It was discovered that CXCL2 promotes HCC metastasis and that its expression is higher in a coculture system with M2 and SMMC7721 cells in addition to HCC tissues [179]. Conversely, in order to ascertain if the reduction in CXCL2 observed in HCC [180, 181] was regulated by DNA methylation, the CXCL2 levels of HCC cell lines were elevated after treatment with the DNA demethylating agent 5-aza-2′-deoxycytidine [181].

### Histone modification

Many liver illnesses, including HCC, revolve around changes in the pattern of histone modifications, which include methylation, phosphorylation, acetylation, glycosylation and others [182]. Most importantly, more and more research is focused on histone modifications and how they affect cellular signaling and signature gene expression, which in turn affect functional responses of hepatic macrophages and the M1/M2 polarization [183, 184]. Protein arginine methyltransferase 1 (PRMT1) has been confirmed as a critical modulator of inflammatory responses [185] and is required for favoring an anti-inflammatory M2 phenotype through histone H4R3me2a methylation of the PPARγ promoter [186]. Moreover, as a result of c-Myc binding to acetyltransferase p300 and a reduction in recruitment of histone deacetylase 1, PRMT1-dependent arginine methylation is essential for c-Myc function during M2 differentiation [185]. One crucial mechanism in the progression of alcohol-associated tumors is the activation of the PRMT1–IL-6–STAT3 axis, which is correlated with PRMT1 expression in TAMs and STAT3 activation in human and animal HCC tissues [187]. These findings could be helpful in evaluating the pathologic processes of HCC and point to PRMT1-dependent M2 polarization as being caused by dysregulation of histone modifications. There is a crucial tumorigenic phase where the tumor site inflammation in the liver can significantly impact the biological activity of a malignant tumor [188].

## Anti-tumor compounds and macrophages in HCC

There has been significant improvement in field of cancer therapy by development of precision medicine. Since macrophages are critical players in TME in HCC, their inhibition by drugs can lead to reduction in tumor malignancy. Emodin is a natural anthraquinone that can be derived from *Rheum palmatum* L and based on in vitro and in vivo experiments, it shows high anti-tumor activity [189–191]. The excessive response of M1 and M2 polarized macrophages is suppressed by emodin and it can affect macrophage homeostasis in human malignancies [192]. Emdoin has been shown to suppress M2 polarization of macrophages in alleviation of inflammation [193]. In treatment of HCC, emodin is able to affect macrophage polarization. Emodin increases levels of miR-26a and then, suppresses TGF-β1 signaling in mediating M1 polarization of macrophages and decreasing HCC progression [194]. Cantharidin is another anti-tumor compound in treatment of HCC that suppresses STAT3 and PI3K/Akt pathways in reducing survival rate and colony formation of tumor cells [195]. Moreover, cantharidin stimulates apoptosis and G2/M arrest in HCC cells [196]. Cantharidin enhances miR-214 levels in suppressing β-catenin for increasing M1 polarization of macrophages. Moreover, cantharidin inhibits STAT3 pathway in macrophages in HCC [197]. Hence, changing macrophage polarization affects progression of HCC cells.

YAP is a newly discovered factor in HCC whose upregulation can result in sorafenib resistance in HCC cells by avoiding ferroptosis [198]. USP10 is capable of increasing levels of YAP and promoting its stability in accelerating progression and growth rate of HCC cells [199]. As an anti-cancer agent, ligustilide can reduce expression level and nuclear transfer of YAP to suppress IL-6 secretion in impairing M2 polarization and shifting macrophage polarization to M1 in decreasing HCC progression [200]. Since nature is a rich source of anti-tumor agents, various anti-tumor compounds have been introduced from nature in treatment of HCC. Zoledronic acid (ZA) increases cyclin A expression and diminishes cdc2 levels in decreasing proliferation and mediating S arrest in HCC cells [201]. The transcatheter arterial chemoembolization (TACE) is an advantageous therapy for HCC, but its potential needs to be improved. ZA in combination with TACE suppresses angiogenesis via VEGF downregulation and inhibits the infiltration of TAMs in TME in reducing HCC progression [202].

Immunotherapy has been emerged as one of the most promising candidates in treatment of HCC [203, 204] that aims in educating anti-tumor immunity. However, the efficacy of immunotherapy depends on TME and it can be compromised by changes in this environment. The cells with immunosuppressive function that infiltrate into TME can lead to changes in anti-tumor response and decrease potential of cancer immunotherapy [205, 206]. TAMs infiltration in TME is a problem for cancer immunotherapy. *Terminalia bellirica* (Gaertn.) Roxb. (TB-TF) has been shown to affect education of macrophages in TME. TB-TF suppresses HC progression in vivo and induces re-education of macrophages from M2 polarization to M1 polarization that is accompanied with enhancement in levels of TNF-α, IL-1β and iNOS and reduction in Arg-1 levels. Moreover, TB-TF can increase the infiltration of T cells in TME and promotes anti-tumor function of cytotoxic CD8+ T cells [207]. The levels of pro-inflammatory factors are vital for changes in polarization of macrophages and reducing their secretion can affect macrophage polarization in TME [208]. Quercetin is a natural anti-tumor compound in cancer therapy and it mediates autophagy-induced apoptosis in reducing HCC malignancy [209]. Moreover, quercetin is able to inhibit proteasome activity in HCC via suppressing MEK1/ERK1/2 axis [210]. Quercetin suppresses NF-κB axis and diminishes levels of TNF-α, IL-6 and IL-17A to enhance M1 polarization of macrophages [208]. When polarization of macrophages changes into M1, the anti-tumor responses in HCC increase. It has been reported that bufalin is able to reduce upregulation of p50 to increase accumulation of p65–p50 heterodimers in nucleus and mediates a predominance over p50 homodimer in nucleus, resulting in NF-κB induction that mediates M1 polarization of macrophages and increases infiltration of T cells in TME for enhancing anti-tumor immunity [117]. One of the controversies is related to regulation of NF-κB by anti-tumor compounds, which is that both inhibition and induction of this critical pathway have been observed.

In addition to phytochemicals, synthetic drugs have also been used for regulating macrophage polarization in TME of HCC. When sorafenib is applied for HCC therapy, it causes proptosis in macrophages and this is beneficial for improving anti-tumor activity of natural killer cells in reducing HCC malignancy [211]. Upregulation of MAPK pathway leads to increased M2 polarization of macrophages and reduction in anti-tumor immunity in HCC. However, when regorafenib is applied in treatment of HCC, it causes inhibition of p38 MAPK axis to downregulate expression level of its downstream targets including Creb1 and Klf4 to suppress M2 polarization in enhancing anti-tumor immunity in HCC [212]. Therefore, significant effort has been made in using anti-tumor compounds to suppress TAMs in TME of HCC [213, 214]. Another advancement in cancer therapy is application of nanostructures in regulating macrophage polarization in HCC. A combination of doxorubicin-loaded liposomes and clodronate-loaded liposomes has been utilized in HCC therapy and after intra-spleen injection, it was found that they are able to deplete macrophages and reduce number of hepatic CD68 + macrophages that these changes in TME can result in significant reduction in HCC progression [215]. Table 1 provides a summary of macrophage role in HCC progression and therapeutic approaches.

**Table 1:** The role of macrophages in TME of HCC.

Molecular pathway	Remark	Ref
HMGA1/NF-κB/CCL2	The upregulation of NF-κB/CCL2 axis by HMGA1 to increase progression of HCC through macrophage recruitment	[236]
HDAC2/ACKR3	HDAC2 increases expression level of ACKR3 through STAT1 to induce M2 polarization	[237]
APOC1	Downregulation of APOC1 increases M1 polarization of macrophages	[238]
miR-142-3p	Propofol suppresses tumorigenesis in HCC through transfer of miR-142-3p from macrophages to tumor cells through macrovesicles	[239]
IRE1α	Genipin reduces IRE1α expression on TAMs to suppress HCC progression	[240]
HMGB1/IL-6	HMGB1 induced reprogramming of macrophages to secrete IL-6 for tumorigenesis in HCC	[241]
STAT3	TAMs increase STAT3 expression in cancer progression acceleration	[76]
CXCR4/ERK	Extracellular ubiquitin evokes M2 polarization of TAMs through stimulation of CXCR4/ERK axis	[242]
CCAT1/Let-7b/HMGA2	TAMs increase HCC progression through stimulation of CCAT1/Let-7b/HMGA2 axis	[243]
–	Deletion of Dicer in hepatocytes stimulates M1 polarization	[244]
miR-98/IL-10	miR-98 reduces IL-10 expression in suppressing impact of TAMs in increasing proliferation and metastasis	[245]
H19/miR-193b/MAPK1	Macrophages increase H19 expression to increase MAPK1 expression through mIR-193b downregulation in increasing carcinogenesis	[246]
miR-125a/b/CD90	CD90 modulation by miR-125a/b suppresses TAMs	[247]
miR-98	miR-98 inhibits function of TAMs in EMT induction	[248]
miR-155	miR-155 stimulates M2 polarization of macrophages to increase tumorigenesis	[249]
PART1	The presence of lncRNA PART1 in extracellular vesicles derived tumor cells increases M2 polarization of TAMs	[250]
miR-26a/M-CSF	miR-26a reduces M-CSF to suppress macrophage recruitment	[219]
COX-2	LncRNA COX-2 suppresses M2 polarization of macrophages	[166]
HMMR-AS1/miR-147a/ARID3A	HMMR-AS1 increases ARID3A expression through miR-147a inhibition to induce M2 polarization	[251]
LINC00662/Wnt	LINC00662 increases Wnt expression to induce M2 polarization	[252]
Circ_0074854	Silencing circ_0074854 impairs M2 polarization of TAMs	[253]
miR-452-5p	Exosomal miR-452-5p stimulates M2 polarization and increases tumorigenesis	[254]

## Strategies for the regulation of macrophages in HCC

### Monocyte recruitment suppression

The depletion of TAMs is vital for impairing the progression of HCC [216]. The recruitment of TAMs to TME can be provided by CCL2/CCR2 axis and one of the major sources of CCL2 is KCs [217]. Therefore, it is possible that the KCs are involved in the process of attracting and training macrophages that are related to monocytes. CCL2 is a prognostic factor in HCC and suppression of CCL2/CCR2 axis can suppress TAMs toward M2 polarization and impair the progression of murine liver tumor model through induction of T cell-mediated anti-cancer immune reactions [31]. In this case, a CCL2-neutralizing antibody has been introduced to decrease the levels of inflammatory myeloid cells and suppress the expression of IL-6 and TNF-α in HCC model [218]. The miR-26a can reduce the levels of M-CSF via PI3K/Akt control to reduce the recruitment of TAMs into TME infiltration [219]. The glypican-3-targeting antibodies can suppress the recruitment of M2 polarized macrophages to impair the progression of HCC and they have been used in phase I clinical studies [220, 221].

### TAM elimination

The combinatorial studies have focused on the regulation of TAMs for cancer immunotherapy [222]. Sorafenib [122] has been suggested as an oral multi-kinase inhibitor confirmed for the treatment of HCC that can impair the function of polarized macrophages in the induction of EMT in HCC and reduction in their migration [223]. Moreover, ZA has been shown to accelerate apoptosis in TAMs [224, 225]. ZA-mediated TAM suppression is able to increase the function of sorafenib in cancer therapy, angiogenesis inhibition and lung metastasis suppression in HCC [226]. Moreover, ZA is able to decrease infiltration of TAMs and angiogenesis in the rat models of HCC [202].

### TAM reprogramming

The reprogramming of TAMs has also been suggested for impairing the progression of HCC. Baicalin has been shown to increase M1 polarization of macrophages and enhance the generation of pro-inflammatory cytokines [227]. 8-Bromo-7-methoxychrysin is another factor that can impair the expression of CD163 (the maker of M2 polarized macrophages) [228]. PLX3397 is able to suppress the growth of xenograft model through enhancing M1 polarization of macrophages and the blockade of CSF-1R can reduce the growth of tumor [229].

## Prognostic function of macrophages in HCC

Recently, it was suggested that CD68 macrophages could be used as a prognostic factor in HCC. However, the results of various studies are inconsistent. In a meta-analysis of 20 observational studies with a total of 4297 patients, TAMs were proposed as independent predictive indicators and therapeutic targets for HCC, with different effects observed for different TAM subtypes. A high density of CD68 TAMs in either the intratumor (IT) region or peritumor region was associated with poor overall survival. The authors found that a high density of CD68 TAMs in the IT region was also associated with high AFP levels, large tumor size, absence of encapsulation, presence of vascular invasion and a higher tumor-node-metastasis (TNM) stage. Additionally, OS was associated with a high CD163 macrophage density in serum. There was a correlation between a high CD204 TAM density in the IT region and a poor overall survival rate, and between a high CD206 TAM density in the IT region and both poor overall survival and disease-free survival. Conversely, a high density of CD169 TAMs in the IT region was associated with favorable OS [230]. It is important to note that using TAMs within the TME as a single biomarker may not be sufficient to fully elucidate their role. Several other factors should be taken into account when assessing the use of TAMs as a prognostic factor [231].

## Conclusion and remarks

The clinical management of HCC has been a challenging issue for physicians due to malignancy of these cells and one of the problems is low and poor prognosis of patients. Due to involvement of different factors in pathogenesis and progression of HCC, studies have focused on the specific and important factors in HCC carcinogenesis. The crosstalk of HCC cells with other cells in the TME can change tumorigenesis process and since TAMs are abundant and frequently observed in this space, current review dedicated to understanding function of these factors in tumor progression. The mission of this manuscript was to show how TAMs participate in tumorigenesis in HCC and how they enhance growth and invasion of HCC cells that can cause poor prognosis of patients at clinical level. Moreover, proliferation rate of HCC cells can be facilitated by function of TAMs through stimulating glycolysis. More studies have shown that TAMs reduce response of HCC cells to therapy, especially chemotherapy and if there is therapy failure in patients, researchers can target TAMs in resolving this condition. Macrophages have ability of secreting exosomes that can mediate communication among cells in TME by transferring important bioactive molecules, especially ncRNAs. The mechanisms responsible for M1 to M2 polarization of macrophages in HCC have been highlighted that ncRNAs are the most important regulators and then, anti-cancer compounds for targeting TAMs in HCC have been employed. Understanding the function of TAMs in HCC is also of importance in the clinical studies. This has been also indirectly evaluated in the clinical level. Proteoglycan glypican-3 is essential for cell proliferation, differentiation and migration; it binds to cell surfaces [232]. As a tumor-derived carcinoembryonic antigen, its high expression in HCC tissue is associated with a poor prognosis. For example, glypican-3 expressions are related to the overexpression of CSF-1, CCL3 and CCL5 in an HCC xenograft model [233], all of which are chemokines that enhance the recruitment of TAMs. Glypican-3 antibodies showed encouraging effects in 13 and 20 patients, respectively, in modest phase I trials for advanced HCC. Patients treated with advanced HCC (Child-Pugh A or B cirrhosis) had a threefold longer median time to progression after receiving the antibody, and it is well-tolerated [234, 235]. Currently, no phase II trials are registered for glypican-3 antibodies in HCC. Hence, more clinical studies will shed light on the importance of TAMs in HCC.
